# The Impact of Discrimination on Allostatic Load in Black Middle‐Aged and Older Adults: A Systematic Review and Meta‐Analysis

**DOI:** 10.1002/alz70856_101648

**Published:** 2025-12-25

**Authors:** Alaa Harb, Juliana Nery Souza‐Talarico, Peter Abad, Karen Lawrence, Jihye Lee, Ana W. Capuano, Lisa L. Barnes

**Affiliations:** ^1^ University of Iowa, Iowa City, IA, USA; ^2^ University of Iowa, Iowa, IA, USA; ^3^ The University of Iowa, Iowa City, IA, USA; ^4^ University of Kentucky, Iowa City, KY, USA; ^5^ University of Southern California, Los Angeles, CA, USA; ^6^ Rush University Medical Center, Chicago, IL, USA; ^7^ Rush Alzheimer's Disease Center, Rush University Medical Center, Chicago, IL, USA; ^8^ Rush Alzheimer's Disease Center, Chicago, IL, USA

## Abstract

**Background:**

Discrimination is a pervasive social determinant of health that disproportionately affects Black middle‐aged and older adults. This systematic review and meta‐analysis examined the relationship between discrimination and allostatic load (AL) in this population.

**Methods:**

A systematic search was conducted across multiple databases following PRISMA guidelines. Studies measuring discrimination and AL in Black adults aged 40 and older were included. The ROBINS‐E tool was used to assess study quality. A random‐effects meta‐analysis was performed to estimate the overall effect size.

**Results:**

Six studies (*N* = 5,006) met inclusion criteria. Four studies (66.7%) found significant positive associations between discrimination and AL. Meta‐analysis of five studies (*N* = 4,746) revealed a small‐to‐moderate association between discrimination and AL (Hedges's g=0.321, 95% CI=0.041‐0.601, *p* = 0.025). Current discrimination showed stronger associations with AL compared to past discrimination experiences. Significant heterogeneity was observed across studies (I^2^=94.74%).

**Conclusions:**

This review provides evidence for the relationship between discrimination and AL among Black middle‐aged and older adults. The findings suggest that discrimination's impact on physiological stress may vary by timing and type of exposure. Future longitudinal research is needed to better understand these temporal relationships and inform interventions.

**Keywords**: Perceived Discrimination, Allostatic Load, Black People, Middle Aged

